# Application of comparative biology in GO functional annotation: the mouse model

**DOI:** 10.1007/s00335-015-9580-0

**Published:** 2015-07-04

**Authors:** Harold J. Drabkin, Karen R. Christie, Mary E. Dolan, David P. Hill, Li Ni, Dmitry Sitnikov, Judith A. Blake

**Affiliations:** The Jackson Laboratory, Bar Harbor, ME 04609 USA

## Abstract

The Gene Ontology (GO) is an important component of modern biological knowledge representation with great utility for computational analysis of genomic and genetic data. The Gene Ontology Consortium (GOC) consists of a large team of contributors including curation teams from most model organism database groups as well as curation teams focused on representation of data relevant to specific human diseases. Key to the generation of consistent and comprehensive annotations is the development and use of shared standards and measures of curation quality. The GOC engages all contributors to work to a defined standard of curation that is presented here in the context of annotation of genes in the laboratory mouse. Comprehensive understanding of the origin, epistemology, and coverage of GO annotations is essential for most effective use of GO resources. Here the application of comparative approaches to capturing functional data in the mouse system is described.

## Introduction

The Gene Ontology (GO, The Gene Ontology Consortium 2000, 2015) provides a structured, controlled vocabulary used by a wide range of biological knowledge bases to create annotations that describe a gene product’s function, the overall biological objective of the function, and the cellular location where the function occurs. GO is a widely used biomedical ontology, utilized extensively in data analysis pipelines especially for functional analysis of large datasets. Core methods for providing GO annotations for gene products include curating data from the biomedical literature, inferring information from structural parameters of the gene product, and inferring information based on data mined from homology and phylogenetic assertions to other gene products. Understanding the structure, scope, and origin of GO annotations that summarize current knowledge ensures the best use of GO resources by the research community. Here we focus primarily on the cross-species approach to generating GO annotations, using the Mouse Genome Database-GO curation workflow as an illustrative example. We then provide context for use of cross-species annotations in data analysis applications.

The key elements of a GO annotation are (1) the association of a gene product with a GO term, (2) a statement of the kind of evidence used to make the association (typically an evidence code), and (3) an authority from which the association is made (typically a publication). Here the term ‘gene product’ is used to capture all types of functional entities encoded by genome features including proteins, functional RNAs, and protein complexes. General information about the generation and quality control for GO annotations are discussed in Balakrishnan et al. (2013) and can be found at the GOC website (www.geneontology.org).

The Mouse Genome Database (MGD), the model organism database component of the Mouse Genome Informatics system (MGI; www.informatics.jax.org) (Eppig et al. 2015), makes use of GO terminology to provide functional information about mouse gene products. The MGD curation pipeline proceeds in the environment of curation paradigms developed by the GO Consortium (GOC). These paradigms are employed by all GO annotation providers ensuring consistency in generation and reporting of annotations (http://geneontology.org/page/annotation).

Recently, the GO curation workflow has expanded, so that the curation record can include more details about the context in which the gene product is functioning. This capture of contextual information includes the ability to provide information relative to precise protein forms including isoforms generated by alternative splicing and/or start/stop sites, as well as to protein forms having various post-translational modifications. The capture of contextual information includes describing cell type, anatomical location, time, and other aspects of the functioning of the gene product.

MGD is the authority for providing the comprehensive set of GO annotations for the laboratory mouse to the bioinformatics community. While the majority of mouse annotations are generated within the MGD project, other projects such as the GOA project at UniProt (Huntley et al. 2015) and the PAINT project within the GOC (Gaudet et al. 2011) also generate mouse annotations. These literature-based and sequence similarity-based annotations are imported and integrated into the MGD-authoritative mouse GO annotation file on a weekly basis (ftp://informatics.jax.org/pub/reports/index.html#go). These mouse annotations are then incorporated into the GO data resources such as AmiGO (http://amigo.geneontology.org/amigo, Carbon et al. 2009) and NCBI (NCBI Resource Coordinators 2015), and into other data resources representing current knowledge about mouse genes.

## GO annotation via literature curation

Literature curation remains the primary source for experimentally based knowledge about molecular functions of genes and gene products. Currently, MGD has more than 112,500 curated, literature-based annotations to over 12,300 mouse genes obtained from over 23,200 publications. The data and information captured from the primary literature forms the basis for generation of annotation based on comparative inference. The workflow for obtaining literature and prioritizing papers to curate have been described in detail previously (Drabkin and Blake 2012). In brief, biologist curators with experience in specialized biomedical research fields identify appropriate literature with the assistance of data mining tools, confirm specific entities (such as genes or proteins), and select appropriate GO terms to represent the experimental results reported about these entities. Within MGD, the highest priority for literature curation is given to papers that describe new knowledge about genes for which the GO knowledge capture system has no information. Priority is also given to literature with functional information about genes implicated in important disease processes and to literature for genes whose functional annotations consist only of those inferred through automated processes.

## GO annotation via sequence similarity

Experiment-based annotations form the basis for functional characterization of a gene product. In addition, the use of orthology to infer knowledge about a gene product from experiment-based annotations to a related gene product makes use of the expert knowledge captured and integrated into other model organism databases and resources such as GOA (human and other species, Huntley et al. 2015), RGD (*Rattus*; Shimoyama et al. 2015), FlyBase (*Drosophilia melanogaster*; dos Santos et al. 2015), SGD (*Saccharomyces cerevisiae*; Costanzo et al. 2014), Pombase (*Schizosaccharomyces pombe*; McDowall et al. 2015) WormBase (*Caenorhabditis elegans*; Harris et al. 2014), and DictyBase (*Dictyostelium discoideum*; Basu et al. 2013). Since the development of model organism research systems is a consequence of the utility of different organisms for different types of biological investigation, these similarity-based methods for obtaining functional annotations for mouse gene products bring into the mouse system more global information than has been generated by the mouse experimental system alone. Different assays are employed in different systems, each utilizing the strength of that particular system (e.g., many genes associated with human disease are often studied using cloned cDNA encoding a human protein in cell culture systems). GO curation guidelines provide several sequence similarity-based evidence codes to support the variety of cross-species annotations (see Table 1). This type of comparative inference is used across all of the model organism databases that use GO for functional annotation.

**Table 1:** Sequence-based evidence codes

Inferred from sequence or structural similarity (ISS)
Inferred from sequence orthology (ISO)
Inferred from sequence alignment (ISA)
Inferred from sequence model (ISM)
Inferred from genomic context (IGC)
Inferred from biological aspect of ancestor (IBA)
Inferred from biological aspect of descendant (IBD)
Inferred from key residues (IKR)
Inferred from rapid divergence (IRD)

A complete list of all evidence codes used by GO can be found at http://geneontology.org/page/guide-go-evidence-codes

Within MGD specifically, orthology-based annotations are either captured by MGD curators or generated via semi-automated pipelines. In all cases, only annotations based on experimental characterization are propagated from one species to another, preventing circular annotations between the contributing and receiving resources. Since all GO groups are generating GO annotations via the same paradigm, experimental annotations between these groups are concordant. The standards for generation of orthology data representations between mouse and other organisms is a key to the process. Within vertebrate systems, as with other specific taxonomic groups, assertions of orthology are complicated by gene duplication and paralog divergence events (Sonnhammer et al. 2014).

**Fig. 1:**
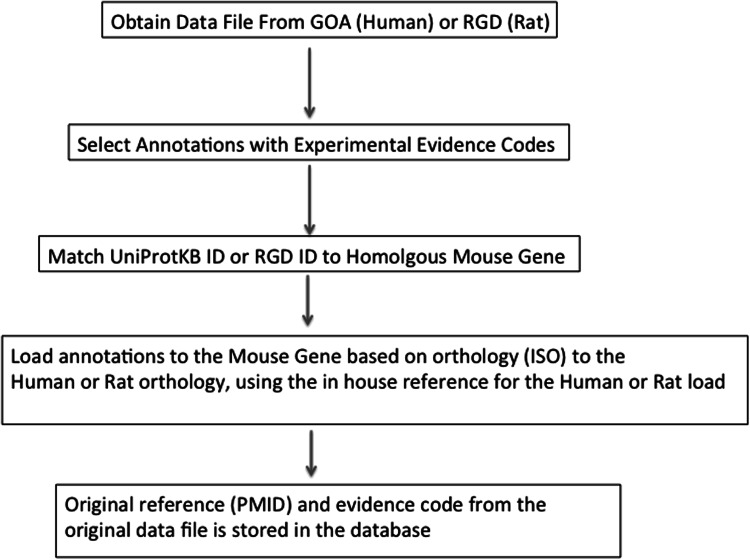
Importing mouse annotations from rat or human genes based on orthology to mouse genes. Each specific load is assigned a specific MGD reference. Since the evidence code is assertion by orthology as determined by MGD, the provider of the annotations is MGD. Annotations are obtained from the designated authorities for GO annotation for human (GOA) or rat (RGD) genes

Rather than the MGD-vetted one-to-one orthology assertions that had been used previously, in 2013, MGD moved to a many-to-many orthology paradigm (see Dolan et al., *Mammalian Genome* this issue) through the use of an external resource, HomoloGene (NCBI Resource Coordinators 2015). Although one-to-one orthology assertions between mouse-human and rat genes still holds for over 90 % of protein-coding genes, MGD can now more clearly represent loci that include a more complex sequence of speciation and gene duplication events. In order to maximize the use of human-mouse orthology sets for comparative genomics in the context of phenotypes or disease, the May 2015 release of MGI also includes the use of HUGO Gene Nomenclature Committee (HGNC) (Gray et al. 2015) mouse–human orthology data. However, currently, HGNC orthology assertions are not used to transfer GO annotation from human to mouse genes. Figure 1 outlines the overall workflow for importing annotations from GOA or RGD based on orthology. Functional annotation of human and rat gene products coming into the MGD system are provided by GOA and by RGD, respectively. As mentioned above, these resources utilize the same GOC annotation guidelines in regard to literature curation of the experimental literature. However, because each species has unique aspects, a variety of rule-based systems have been developed in the MGD system to ensure the assertions result in reasonable predictions. For example, annotations to protein binding or using the NOT qualifier are excluded. Protein binding annotations are excluded because they are created in the context of a specific protein-binding event, something that cannot be reliably transferred between systems. The NOT qualifier is part of annotations where a protein has been demonstrated experimentally to NOT have some property. These cannot be reliably inferred in a cross-species manner. The change to a many-to-many orthology paradigm required careful attention to the development of rules appropriate for the transfer of functional annotation from human or rat experiments to mouse genes, especially in cases of paralogs. Specifically, for any case in which more than one gene per species is in a HomoloGene class, only experimental molecular function and cellular component annotations are transferred as ISOs. In addition, if any member of the class has a ‘NOT’ annotation, annotation to that term is not transferred to any member of the class. At present, the majority of GO annotations in MGD based on orthology/sequence similarity are based on orthology with rat and human genes. A summary of GO annotations based on orthology in MGD is found in Table 2.

**Table 2:** Summary of GO annotations in MGD from literature curation, orthology or electronic pipelines

Annotation method	Total		Genes annotated only by orthology, phylogenetic, or electronic method	
	# Genes	# Annotations	# Genes	# Annotations
Manual curation of experimental literature	11,123	98,944	NA	NA
Orthology transfer methods	11,728^a^	98,987	3728	18,012
Transferred from human (GOA) via orthology	10,515	65,988	3379	14,104
Transferred from rat (RGD) via orthology	4631	29,861	816	3271
Curated by MGI curators	1322	3138	268	637
Phylogenetic methods				
PAINT	4356	19,703	2285	10,841
Electronic pipelines (IEA)	14,653^b^	98,980	5308	35,276
Enzyme Commission (EC)	1690	18,549	692	8848
Swiss-Prot keywords	14,270	55,754	5107	18,369
InterPro	9970	24,677	3346	8060
All annotation methods	24,179	357,251	7219	64,129

Numbers are as of May 5, 2015

^a^Genes can be annotated by multiple orthology methods, so this represents total number of genes annotated by any orthology method

^b^Genes can be annotated by multiple electronic pipelines, so this represents total number of genes annotated by any of them

**Fig. 2:**
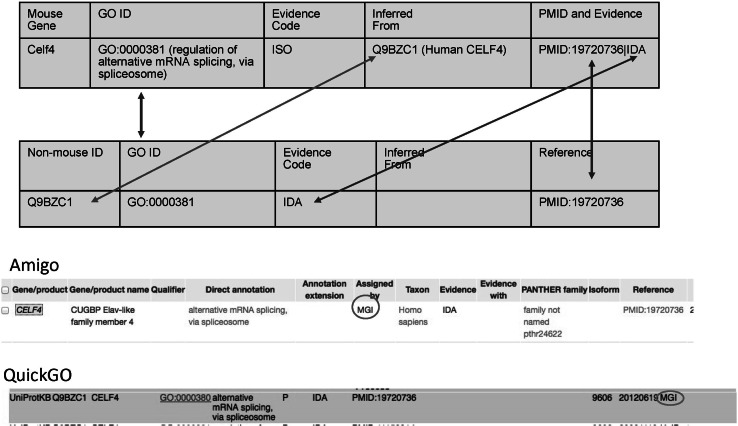
Exporting mouse annotations to non-mouse genes based on orthology. The orthologous non-mouse gene becomes the gene that is annotated by an experimental method described in the publication. The *bottom two panels* depict the non-mouse annotation at either the GOC site (Amigo browser) or GOA (QuickGO)

In addition to obtaining annotations for mouse genes from other species via orthology, MGD also generates experimentally supported orthology-based GO annotations *for* other species during curation of mouse genes. When appropriate, MGD curators may create annotations for the other species when the literature we are curating provides evidence for conservation of function between species. Annotations made by MGD curators using sequence similarity evidence codes (ISO/ISA/ISS) are converted by MGI to annotations to the non-mouse gene based on direct experimental evidence are supplied in GAF format to the GO Annotation (GOA) group at the EBI (European Bioinformatics Institute). For example, as shown in Fig. 2, an annotation for *Celf4* was made by MGI based on orthology. The reference shown (J:73065, GO_REF:0000008) denotes that the annotation is made by orthology. The experimental evidence to base this on is obtained from a publication, which is stored at MGI. The experimentally based annotation for the human gene (CELF4) using that publication and the appropriate evidence code is then output to the GAF file given to GOA. Currently, MGI generates a file of 4877 annotations for over 30 non-mouse species from the ISO annotations MGD provides to the GOA resource. These include data from human, rat, cow, dog, hamster, rabbit, pig, macaque, zebra fish, chicken, and frog.

## Phylogenetically based annotations (PAINT)

**Fig. 3:**
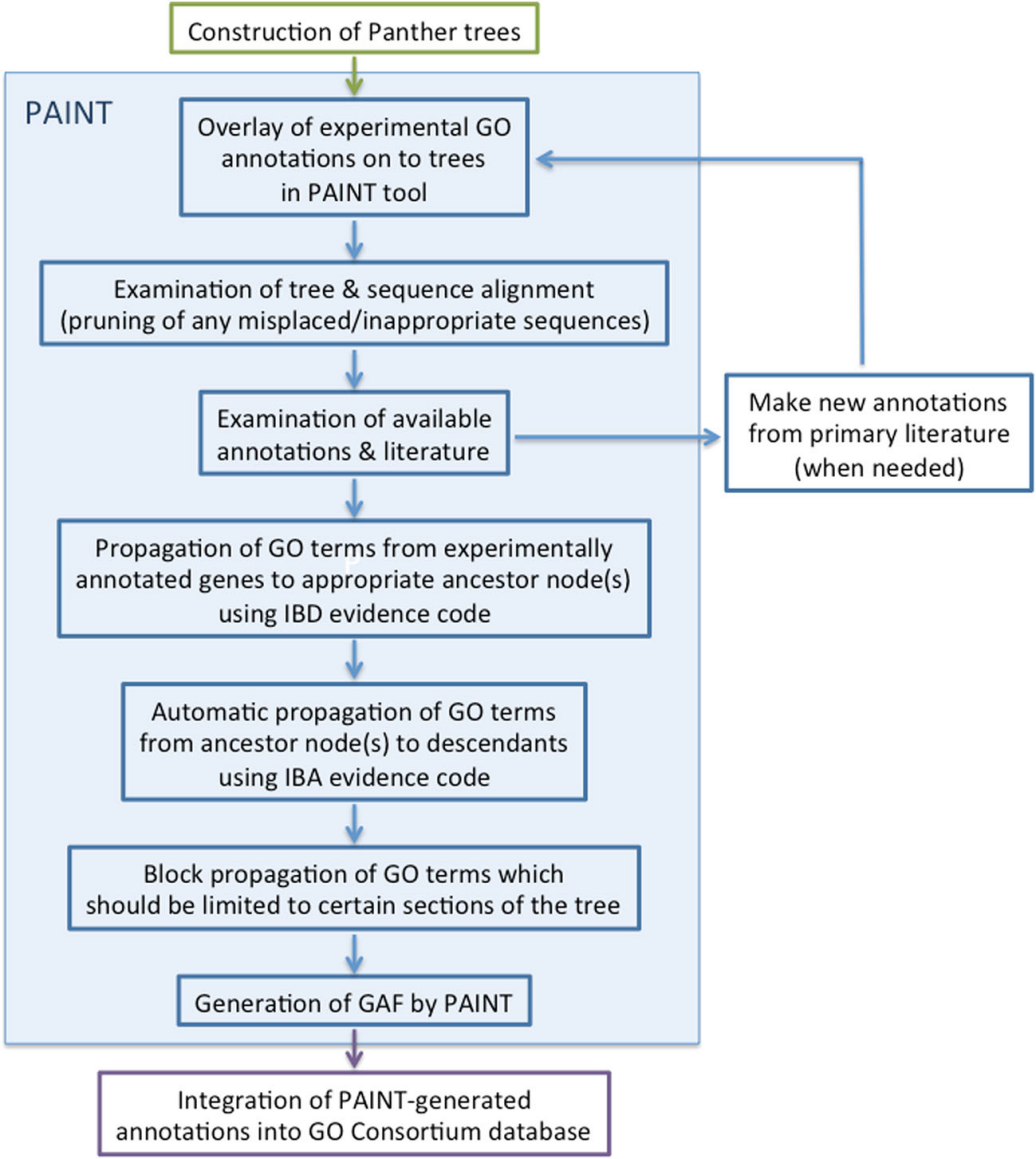
The PAINT tool overlays experimental GO annotations onto externally constructed Panther phylogenetic trees and allows curators to remove any inappropriate or misplaced sequences before propagating annotations. When needed, new annotations can be made which will be included in PAINT once they have been added to the GO Consortium annotation database. The curator can then determine which annotations represent ancestral functions which should be propagated to an ancestral sequence node. PAINT automatically propagates GO terms from the ancestor node to all descendant sequences that are not already annotated to that term experimentally, except where the curator blocks propagation due to divergence in function. The annotations are exported from PAINT and incorporated into the GO Consortium annotation database

In order to facilitate the use of data from mouse and other species in the study of human biology and disease, the GOC has developed a pipeline for generation of cross-species annotations specifically focused on phylogenetic relationships extending across all taxa. These GO annotations are generated within the context of a given protein family as provided by the Panther system (Mi et al. 2009) and are based on the structure of the phylogenetic tree as well as the experimental evidence for characterized members of the family (Gaudet et al. 2011) Annotations asserted by direct experimental evidence, primarily not only from the twelve “reference” model organisms (see Table 3) but also from other experimentally characterized species when available, are overlaid upon a sequence-based evolutionary tree of all proteins in the Panther Tree database. Using the Phylogenetic Annotation and INference Tool (PAINT, Gaudet et al. 2011), curators determine which annotations can be propagated to a common ancestor node of the tree, indicating an ancient conserved function, where those ancestral annotations can be propagated to all descendent members of the tree unless there is evidence that a function has been lost within a branch of the tree (see Fig. 3).

**Table 3:** Twelve model organisms (MODs) used for GO annotations backed by experimental evidence

*Arabidopsis thaliana* (The *Arabidopsis* Information Resource (TAIR))
*Caenorhabditis elegans* (WormBase)
*Danio rerio* (zebrafish; Zebrafish Model Organism Database (ZFIN))
*Dictyostelium discoideum* (dictyBase)
*Drosophila melanogaster* (FlyBase)
*Escherichia coli* (PortEco)
*Gallus gallus* (AgBase)
*Homo sapiens* (human UniProtKB-Gene Ontology Annotation [UniProtKB-GOA] @ EBI)
*Mus musculus* (Mouse Genome Informatics)
*Rattus norvegicus* (Rat Genome Database (RGD))
*Saccharomyces cerevisiae* (*Saccharomyces* Genome Database (SGD))
*Schizosaccharomyces pombe* (Pombase)

The PAINT process is a powerful tool for cross-species annotation. Frequently, experimental work performed in one, or a few, experimental organism(s) is not going to be duplicated in others, and PAINT provides a mechanism to annotate genes from uncharacterized species based on the experimental work that has been done, wherever it may fall within the phylogenetic tree, often allowing use of more specific GO terms than are generated using some of the other annotation transfer pipelines.

### How cross-species annotations aid mouse functional annotation

In a specific example, the nuclear RNA polymerase enzymes have been extensively characterized, supported by experimental data from human and yeast (*S. cerevisiae*). The yeast gene *RPO26,* and also the orthologous human gene POLR2F, is well characterized as a core subunit of three nuclear RNA polymerases, RNAP I, RNAP II, and RNAP III (Cramer et al. 2008; Thomas and Chiang 2006). However, the mouse gene *Polr2f* is not annotated with experimental evidence. PAINT allows the annotation of *Polr2f* with the GO terms specific to all three of these nuclear RNA polymerases. In contrast, the annotation based on the InterPRO domain provides only a general term without the specificity of which nuclear RNA polymerases *Polr2f* is part of, and the annotations generated by sequence orthology with human or rat are incomplete providing only the annotations specific to RNAP II but lacking the RNAP I and RNAP III annotations. Similarly, the axonemal dyneins are well characterized biochemically in *Chlamydomonas reinhardtii* as ATP-dependent microtubule motors, present either in the inner or outer dynein arms (King and Kamiya 2009); comparable biochemical work has not been done in mouse or human. PAINT annotation allows the detailed knowledge of which dyneins are present in which parts of the axoneme to be transferred to many other species based on the phylogenetic relationships. In cases like these, mouse genes receive detailed annotations more specific than that provided by the InterPRO domains, based on the experimental work from other species.

### How mouse annotation helps cross-species annotations

In other cases, detailed work has been done in the mouse that allows transfer of information from mouse to other species. For example, the Doublesex AND MAB-3 Related Transcription (*Dmrt*) factor family is named partly for the *Drosophila* Doublesex gene, where it is involved in sex determination. In the vertebrates, there have been numerous duplications within this family, and some family members have acquired additional functions. *Dmrt3* is involved in the regulation of odontogenesis and specification of ventral spinal cord interneurons (Ahituv et al. 2007; Andersson et al. 2012), *Dmrt2* has been shown to be involved in the regulation of somitogenesis but does not appear to involved in sex determination (Seo et al. 2006; Seo 2007), and *Dmrtb1* appears to have lost DNA binding ability (Murphy et al. 2007), as demonstrated by experimental work in the mouse.

Thus, transfer of experimental annotations via the PAINT tool can increase the annotation coverage across many species, from providing annotations for organisms that lack any experimental work in that area of biology, to filling in a few “missing” annotations for a relatively well-annotated species based on experimental work in a closely related species, e.g., where an enzymatic function has been characterized for a rat gene, but not for the human or mouse orthologs. The PAINT annotation process may also improve the specificity of the GO terms used, allowing more detailed knowledge to be represented in the GO annotations. This level of detailed annotation can provide important information. For example, knowing whether a gene is found in the inner versus the outer dynein arms of the axoneme may allow more accurate assessment of the expected phenotype within the spectrum of primary ciliary dyskinesia (PCD). For the *Dmrt* family of transcription factors, PAINT allows transfer of the different roles of various subclades of the family, including the fact that not all members of this family retain activity in regulation of sex determination.

MGD curators are active members of the PAINT annotation team. Besides making annotations to mouse genes within the MGI system, MGD curators have recently begun to annotate other species directly in UniProt’s GO annotation tool, Protein2GO (Huntley et al. 2015) when such annotations are needed for phylogenetic annotation via PAINT, even when there is not a direct comparison to mouse within the primary reference being annotated that would allow us to use one of our long-standing orthology transfer methods. Annotations to mouse genes made via the PAINT phylogenetic method are imported into the MGD on a weekly basis. A summary of GO annotations from PAINT in MGI is found in Table 2.

## GO annotation via electronic pipelines

Additional MGD automated annotation strategies include data obtained from UniProtKB entries assigned to MGI Genes. These mappings include the Enzyme Commission number assignment, Swiss-Prot keywords, and InterPro. Currently MGI has approximately 99,183 the so-called electronic annotations (IEA) to over 14,650 genes. A summary of these annotations is found in Table 2. Note that because the InterPro mapping entries are manually annotated with terms from the GO (Burge et al. 2012), the annotations based on the mappings are considered of high quality. Mapping files can be found at http://geneontology.org/page/download-mappings#dir.

## Use of GO cross-species/global annotations

Clearly, the generation of orthology- and phylogeny-based annotations brings significant added value to the comprehensive set of GO annotations available for mouse or for any organism. For research groups, including computational biologists and bioinformaticians who incorporate GO annotations in their data analysis streams, understanding the complexities and sources of GO annotations is an important element of effective data analysis (Blake 2013). The primary element in evaluating annotations in a cross-species manner is to review the origination of the knowledge assertion made by the annotation.

## Uses of GO in complex queries

**Fig. 4:**
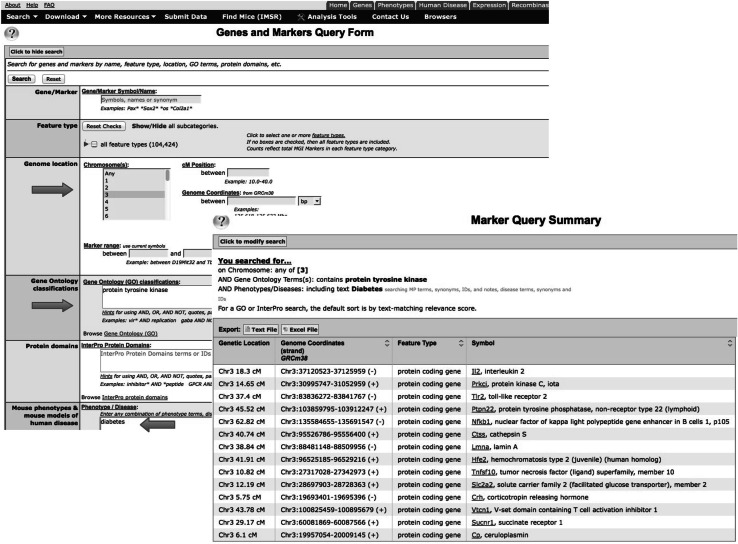
Complex query for mouse genes located on chromosome 3 that are annotated to protein tyrosine kinase activity and are associated with diabetes

**Fig. 5:**
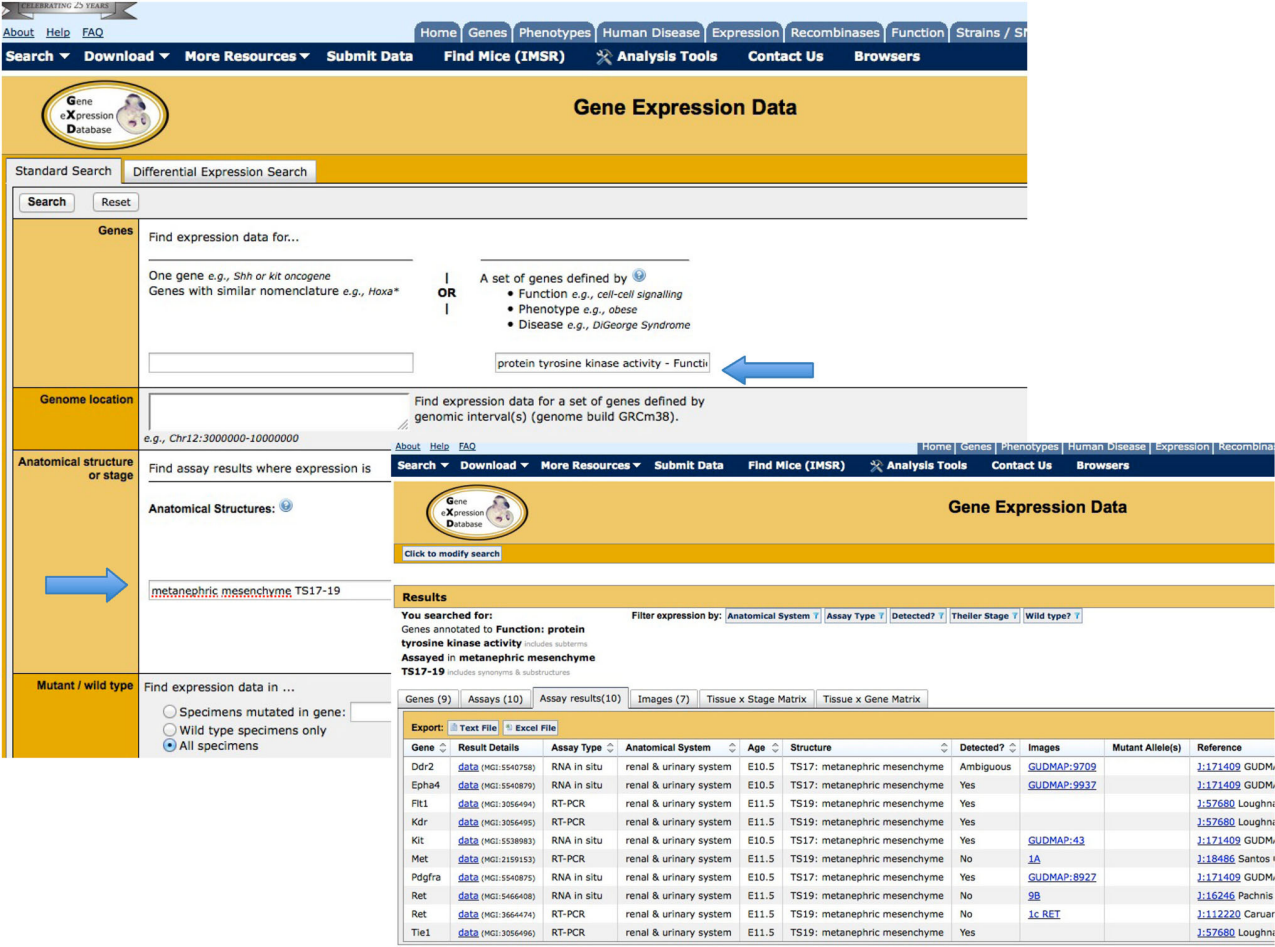
Complex GXD query for mouse genes annotated to protein tyrosine kinase activity and are expressed in Tyler Stages 17–19 metanephric mesenchyme

MGD is a component of the larger Mouse Genome Informatics (MGI) resource. The MGI system is made up of several resources in addition to MGD, such as the Gene Expression Database (GXD) and the Mouse Tumor Database (MTD). MGD curates not just functional information (GO) but also data about mutant mouse alleles, human diseases, and genome structure. GXD curates data on the expression of mouse genes during embryonic development. MTD curates data on the use of mouse models for hereditary cancer. The key paradigm linking these semi-independent curation efforts is achieved by data integration and specifically the fact that all the different types of data are linked to the same gene objects within the database. Thus, GO annotation can be used within the MGD/MGI system for complex queries, such as “show me all genes located on Chromosome 3 that have been annotated to ‘protein tyrosine kinase’ and are associated with Diabetes” (see Fig. 4), or “show me genes annotated to tyrosine kinase that are expressed in metanephric meschyme at Theiler Stage 17” (see Fig. 5).

## Available resources at MGD

Annotations for specific genes can be viewed starting at the Gene Detail page for any one gene. Links provided lead to summaries in tabular, graphical, and textual forms. MGD also provides links to FuncBase for mouse, where one can view computationally predicted GO annotations based on several methods including mutant phenotypes and ‘guilt-by association’ correlations (Beaver et al. 2010). The GO browser can be used to find GO terms as well as a summary of all mouse genes annotated to the term.

All GO annotations in the MGI database, updated nightly, in GAF file format can be found in gene_association.mgi, as well as go_terms.mgi, a file containing a list of all GO terms used at MGI. Annotations in Gene Product Association Data (GPAD, http://geneontology.org/page/gene-product-association-data-gpad-format) will soon be available. The GPAD format is designed to separate annotation object data (synonyms, ids, etc.) from annotation data to reduce redundancy and annotation file size.

There are also several tools available at MGD for use in analyzing GO data, summarized in Table 4.

**Table 4:** Tools available at MGD for GO analysis

Tool	Use	Comments	URL
GO Term Mapper	A tool for analyzing a mouse gene set based on mouse annotations using a method based on the GO Term Finder (Boyle et al. 2004)	Can exclude IEA annotations if desired	http://www.informatics.jax.org/gotools/MGI_Term_Finder.html
GO Slim Chart Tool:	A tool for categorizing a gene set according to a set of high-level GO terms, a ‘GO slim’	Can exclude IEA annotations if desired	http://www.informatics.jax.org/gotools/MGI_GO_Slim_Chart.html
Vlad	A GO Term Finder type tool with a graphical output	Can select annotation set (MGI GO, or user supplied). Can supply reference set and filter on several evidence codes. Output can be graphical, or tabular	http://proto.informatics.jax.org/prototypes/vlad/
MouseMine	An InterMine tool (Kalderimis et al. 2014) that provides access to mouse data for customized queries where the results can be downloaded or reused in subsequent queries	Can use the premade template queries in the FUNCTION section to access GO data in a variety of ways. Results can be further filtered to increase specificity of the query	http://www.mousemine.org/mousemine/begin.do

## Summary

MGD, as a representative member of the GOC, uses a variety of annotation strategies to provide the best possible annotation set for mouse genes and to contribute to the annotation of the other reference genomes. When genes are experimentally characterized in the mouse, we strive to represent this work with experimental GO annotations based on the published literature. However, some genes have not been experimentally characterized in the mouse. Some of these genes may never be fully experimentally characterized in the mouse, but highly conserved, homologous genes have been well characterized in another experimental system, and the findings may be applicable to mouse, e.g., RNA polymerase genes have been extensively characterized with human constructs and in *S. cerevisiae,* and axonemal dyneins have been experimentally characterized primarily in *Chlamydomonas reinhardtii*. For genes where there is experimental work on the orthologous gene in a closely related vertebrate such as rat or human, we are able to use our orthology-based sequence similarity annotation pipelines to provide informative GO annotations about the mouse genes. In other cases, where the experimental work has been done in an organism that is more distantly related and may not have a clear orthology with mouse, being able to make experimental annotations directly for the experimentally characterized organism allows us to use the PAINT tool to utilize the phylogenetic relationships to make informative annotations for evolutionarily related genes, from mouse and many other species. Thus, using direct experimental annotations, as well as a variety of orthology- and phylogeny-based tools to utilize experimental work from many species, MGD strives to provide a comprehensive set of annotations for all mouse genes and also contributes to the improvement in the annotations of genes from other species.
